# The Resurrection of a Child—an Incident of Hahnemann’s Practice*Editors Homœopathic Physician:—The following sketch is translated from Paris *Figaro*, 19th March, 1887, being an extract from a volume of “Memoirs” then in process of publication. The author, Ernest Legouvé, Vice-Doyen of the French Academy, evidently appreciated the life, character, and works of Samuel Hahnemann. Knowing well what interest attaches to all which concerns the master of our art, I have essayed a translation, which I offer you in the belief that others will read it, and share the pleasure it affords me.Very truly yours,Translator.

**Published:** 1889-02

**Authors:** Ernest Legouvé


### THE RESURRECTION OF A CHILD*—AN INCIDENT OF HAHNEMANN'S PRACTICE.

Samuel Hahnemann was one of the grand innovators of the nineteenth century. He inaugurated, about the year 1835, a medical revolution the effects of which are still felt. I do not discuss the system, I only state the fact.

A happy chance, for which I can never be grateful enough, put me in communication with him at the time when his reputation was most glorious. I did not fail to profit by this acquaintance, and the description of some incidents which passed during our intimacy may serve to make known this man, so extraordinary and superior.

My daughter, aged four, was dying; our doctor, Physician of l'Hôtel-Dieu, the Docteur R., had declared in the morning to one of our friends that she was irretrievably lost.

We were watching, her mother and I, as we believed, for the last time, by her cradle; two of our friends, Schœlcher and Gouboux, watched with us; there was also a young man in full evening dress, whom we had not known three hours before. He was one of the most distinguished pupils of M. Ingres, and was called Amaury Duval.

We had desired to preserve at least a souvenir of the dear little creature whose loss we already deplored, and Amaury, persuaded by Schœlcher, who had gone to seek him at a ball, had consented to come and make her portrait.

When this charming artist (he was then twenty-nine years of age) entered, troubled and moved, in the midst of our despair, we little expected that some hours later he would render us the greatest service that we had ever received, nor that we would owe to him not only the counterfeit presentment of our daughter, but her life as well. He placed at the foot of the cradle, on a

---

* Editors Homœopathic Physician:—The following sketch is translated from Paris *Figaro*, 19th March, 1887, being an extract from a volume of "Memoirs" then in process of publication. The author, Ernest Legouvé, Vice-Doyen of the French Academy, evidently appreciated the life, character, and works of Samuel Hahnemann. Knowing well what interest attaches to all which concerns the master of our art, I have essayed a translation, which I offer you in the belief that others will read it, and share the pleasure it affords me.

Very truly yours, Translator.

high chair, a lamp whose rays fell upon the face of the child. Here eyes were already closed, all movements had ceased. Her thin hair laid in disorder on the pillow, which was not whiter than her cheeks; but infancy has in itself such a charm that approaching death seemed to lend but an additional sweetness to her face.

Amaury passed the night at his sketch, often wiping his eyes to prevent the tears from blotting his paper. At daybreak the portrait was finished. Sympathy aiding, his genius had achieved a masterpiece.

At the moment of quitting us, when we were mingling our tears and our thanks, he said, suddenly, " But, since your doctor declares your child beyond help, why do you not seek the aid of this new method, which commences to make such a stir in Paris; why do you not seek the aid of Hahnemann?"

"He is right!" exclaimed Gouboux. "Hahnemann is my neighbor, he lives Rue de Milan, just opposite; I do not know him, but that does not matter; I will go and I will bring him back with me."

Arrived at the house of Hahnemann, he finds twenty persons in the waiting-room. The domestic explains to him that he will have to wait. " Wait!" exclaims Gouboux; " the daughter of my friend is dying, the Doctor must come with me."

" But, sir!" exclaims the domestic.

"Yes, yes, I understand; I am the last. What matter? The Scriptures affirm that the last shall be first." Then turning toward the Doctor's waiting patients:

" Is it not so, ladies? Am I not right? You do, indeed, wish to give me your places, do you not?" And, without waiting a reply, he goes direct to the door of the consultation-room, opens it, enters in the midst of a consultation. " Doctor," says he to Hannemann, "that which I have just done is contrary to all rules, but you must quit all and come with me. It concerns a little girl of four years, daughter of my friend; she is dying. She will die if you do not come. You cannot let her die; it is impossible."

The invincible charm of the manner of Gouboux operated as usual, and an hour later Hahnemann and his wife arrived at the bedside of our little patient.

My mind distracted by grief, and my head reeling from loss of sleep, I fancied, at the first glance at Dr. Hahnemann, that I was regarding one of the personages newly descended from the pages of some of Hoffman's fantastic tales.

Small of stature, but robust and firm of step, he advanced, enveloped in a pelisse of fur, and supported by a heavy cane, gold-mounted. He was nearly eighty years old, his head was admirable; his white, silky hair was thrown back and neatly arranged in curls around his neck (nape).

The centre of the eye was of a profound blue, with a circle almost white around the circumference of the pupil; the mouth was imperious and commanding, the lower lip slightly advanced; the nose was curved like the beak of an eagle. As he entered, he went straight to the cradle of the child, cast upon the patient a piercing glance, and demanded the details of its illness, to which he listened, without once withdrawing his gaze from his patient.

As he listened, his cheeks became flushed, the veins of his forehead swelled, and he exclaimed, in a tone of anger, "Throw, for me, out of the window all that mass of drugs and vials which I see there; carry this patient out of this chamber, change everything, pillows, sheets, etc., all; give her as much water as she will drink; they have thrown live coals into her body; we must first extinguish the fire."

We hazarded the observation that this change of linen, change of temperature, etc., might prove dangerous for the child.

"That which is mortal for her," he replied, with a tinge of impatience, "is this atmosphere and these drugs; take her into the drawing-room, I will return this evening. And above all, give her water, water, water!"

He returned in the evening, but not till the following day did he commence medication. At each visit he contented himself by saying, "Yet another day gained."

The tenth day the peril again became suddenly imminent. The child was cold to the knees. He arrived at eight in the evening, seated himself near her, and remained there, motionless, during a quarter of an hour, watching the child with the air of a man who was the prey of the most poignant anxiety. At last, after having consulted his wife (who always accompanied him), he gave us a medicine, saying, "let her take this, and watch well her pulse; see if it be not stronger an hour from now."

At eleven o'clock, while I was holding the wrist of the child, I suddenly seemed to be sensible of a slight modification of the pulse-beat. I called my wife, then Gouboux, then Schœlcher. We felt the pulse in turn, counted the pulsations, compared our counts, one scarcely daring confirm the observation of the other, until after some minutes we discovered so marked an increase of strength in the pulsations that we embraced each other joyfully, though tearfully. Toward midnight my friend Chretien Uhran entered the chamber, he came toward me, and said, with an air of profound conviction, "My dear M. Legouvé, your daughter is saved."

I replied, "She is slightly better, but the distance from that to a cure is very great."

"I tell you that she is saved," he said, and approaching the cradle where I watched alone, he kissed her on the forehead and departed.

Eight days later the child was fully restored to health.

The manner in which Hahnemann conceived his system of medicine paints his character at a single stroke. Was it on his part calculation? interest? desire of renown? or a conception purely scientific? No! His system came from his heart! Physician of the first order, at the head of one of the richest *clientèles* of Germany, he asked one day the assistance of one of his brother practitioners for his last child, gravely ill; the case was indeed grave, the remedies ordered were heroic, energetic, violent, painful; moxas, cuppings, bleedings, etc.

Suddenly, after a night of terrible suffering for the child, Hahnemann, seized with pity and horror, exclaimed: "No, it is not possible! God has not created these dear little beings in order that they should be submitted to such tortures. No! I *will* not be the executioner of my children."

It was then that, aided by his long and profound studies of chemistry that he set himself to the task of seeking a new physic. It was then that he commenced the construction of this medical system of which paternal love was at once the foundation and the incentive.

Behold the man! As he was then so he was always. The strong structure of his face, his square jaws, the almost continual movement of the alæ nasi, the mobile corners of the mouth slightly depressed by age; everything in him breathed conviction, passion, authority.

His conversation was like his person, original and unique.

"Why," said I to him one day, "do you prescribe, even in health, the continual and habitual use of water as a beverage?"

He replied: "Of what use are the crutches of wine when one is sound of limb."

(*A quoi bon quand on est ingambe les béquilles du vin?*)

Again, it is from him that I heard this phrase, so strange if taken in its absolute sense, but so profoundly true for those who know and understand. "There are no diseases, there are only diseased persons."

(*Il ny a pas de maladies, il y a des malades.*)

His religious faith was not less lively than was his medical.

On arriving at his house one day in spring-time, I remarked: "O M. Hahnemann! what a beautiful day!"

"All the days are beautiful," he replied, with a voice calm and grave. Like Marcus Aurelius, he lived in the bosom of the general harmony.

My daughter being cured, I showed him the beautiful design of Amaury Duval. He contemplated for a long time and with emotion, this image of his little resuscitated patient, as she was at the time he had first seen her, so near death; he then asked for a pen, and wrote on the margin of the sketch, these words:

"God has blessed her and saved her. SAMUEL HAHNEMANN."

His portrait would be incomplete if I did not add that of his wife, who never quitted his side. In his studio she was always seated near his desk at a little table, where she worked like himself. She assisted at all consultations, no matter what the malady, or of which sex the patient. She wrote all the indications (symptoms?) of the malady, gave her opinion in German to Hahnemann, and prepared the medicines.

If, in exceptional cases, he made visits at the houses of patients, she accompanied him always.

This singular fact remains to be recorded, viz., that Hahnemann was the third illustrious old man to whom she had attached herself as wife and helpmate.

She had commenced with painting, passed to literature, and finished with medicine.

At the age of twenty-five or thirty years Miss d'Hervilly (her maiden name) was beautiful, tall, elegant, with a clear, fresh complexion; her face framed in a mass of waving, curling, blonde ringlets, her eyes were blue, small, and with a regard as piercing as black eyes generally have. At this age she became the wife of a celebrated painter, a pupil of David, a M. L——. In espousing the painter she married also his paintings, and it is said that she earned the right to sign her own name to more than one of his works, even as later she signed the prescriptions of Hahnemann. After the death of M. L—— she turned toward poesy, as represented by a poet of seventy years, for, the farther she went, the more she loved the aged. This poet was M. A——. She then threw herself with as much ardor into poetry as formerly she had into the great historical paintings of M. L——.

M. A—— being dead, the *septuagénaires* sufficed her no more, and she married Hahnemann in his eightieth year! becoming at a single step as revolutionary in medicine as she had formerly been classical and orthodox in painting and literature. Her "*culte*" was almost a fanaticism. One day I complained in her presence of the thieving propensities of one of my domestics, whom we had been obliged to dismiss. "Why did you not tell me sooner," said she, quickly and with animation, "we have medicine suitable for such cases."

It would, however, be unfair not to admit that she was of an intelligence really rare, with the soothing address of a born nurse. None knew better than she how to invent the thousand and one devices to comfort the suffering. And more, she united in herself the pious ardor of a Sister of Charity with the thousand fascinations of a woman of the world. Her care for Hahnemann was past praise. He died, as he wished, surrounded by all her tender cares.

Until his eighty-fourth year he lived the most eloquent demonstration of the goodness (bonte) of his doctrine. Not a weakness, not a lack or tremor of intelligence or memory! His diet was simple, but without any affected rigor. He never drank either pure wine or water; a few spoonfuls of champagne in a carafe of water, this was his only beverage. For bread, he ate every day a "*baba*."*

"My old teeth," said he, "find this bread more tender."

During the summer on every clear evening he took a little stroll as far as "l'Arc de Triomphe," stopping habitually at Tortini's to take an ice.

One morning on awaking, he found himself less well than usual; he prescribed for himself, and remarked to his wife: "If this medicine does not have the desired effect, the matter is grave."

---

*"Baba" is a species of bread where fruits are introduced. It is of Polish origin, and is supposed to have been introduced at Paris by King Stanislaus, of Poland.

This "bread" is made fresh every day, and resembles much the ordinary "petit poins" of Paris.

Ingredients: currants, raisins, citron, saffron, cream.

The next day his strength diminished, and twenty-four hours later he died peacefully, recommending his soul to God.

His death was to me a great sorrow; few men other than he have impressed me as he did with the idea of a superior being. You ask, then, "why have you abandoned his doctrine?" I answer, "Through admiration for him."

It seems to me that to follow Homœopathy it demands more than confidence; it demands faith. The theory of infinitesimal doses shocks so rudely our good sense that one must believe blindly in the man in order to believe in the thing. So, Hahnemann having disappeared, my "*culte*" fell with the object of my *culte*, and his successors appeared to me to be so far beneath him that, little by little (a new friendship aiding), I returned to the medical religion of my fathers, under which religion I will die.

I owed, nevertheless, this homage to Hahnemann, and my *ex voto* can only be the more valuable from having been offered by an apostate. ERNEST LEGOUVÉ.

